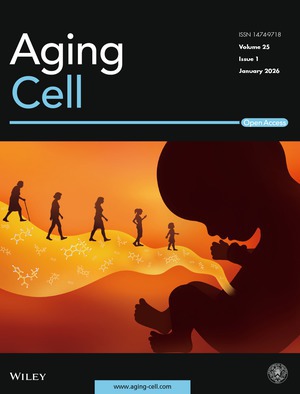# Additional Cover

**DOI:** 10.1111/acel.70413

**Published:** 2026-02-15

**Authors:** JiaYu Liu, Shuai Jiang, YanYan Shen, RuiBo Wang, Zhi Jin, YanQing Cao, JinLiang Li, YanHong Liu, Qi Qi, Yue Guo, YunYing Wang, BoYang Xie, JunCheng Li, AiPing Cao, Yao Wang, ChunYan Yan, QiuYing Han, YingJie Zhu, Jing Peng, FangTing Dong, Xin Pan, XinHua He, Tao Zhou, AiLing Li, Kun He, Na Wang, WeiNa Zhang, Qing Xia

## Abstract

The cover image is based on the article *Human Umbilical Cord Plasma Metabolomics Uncover Potential Metabolites for Combating Aging* by JiaYu Liu *et al.*, https://doi.org/10.1111/acel.70295.